# Text-Mining Approach to Identify Hub Genes of Cancer Metastasis and Potential Drug Repurposing to Target Them

**DOI:** 10.3390/jcm11082130

**Published:** 2022-04-11

**Authors:** Trishna Saha Detroja, Hava Gil-Henn, Abraham O. Samson

**Affiliations:** 1 Cell Migration and Invasion Lab, The Azrieli Faculty of Medicine, Bar-Ilan University, Safed 1311502, Israel; 2 Drug Discovery Lab, Azrieli Faculty of Medicine, Bar Ilan University, Safed 1311502, Israel; avraham.samson@biu.ac.il

**Keywords:** cancer metastasis, drug repurposing, text-mining, hub genes

## Abstract

Metastasis accounts for the majority of cancer-related deaths. Despite decades of research, the prevention and suppression of metastasis remain an elusive goal, and to date, only a few metastasis-related genes have been targeted therapeutically. Thus, there is a strong need to find potential genes involved in key driver traits of metastasis and their available drugs. In this study, we identified genes associated with metastasis and repurposable drugs that potentially target them. First, we use text mining of PubMed citations to identify candidate genes associated with metastatic processes, such as invadopodia, motility, movement, metastasis, invasion, wound healing, EMT (epithelial to mesenchymal transition), and podosome. Next, we annotated the top genes involved in each process as a driver, tumor suppressor, or oncogene. Then, a total of 185 unique cancer genes involved in metastasis-related processes were used for hub gene analysis using bioinformatics tools. Notably, a total of 77 hub genes were identified. Further, we used virtual screening data of druggable candidate hub genes involved in metastasis and identified potential drugs that can be repurposed as anti-metastatic drugs. Remarkably, we found a total of 50 approved drugs that have the potential to be repurposed against 19 hub genes involved in metastasis-related processes. These 50 drugs were also found to be validated in different cancer cell lines, such as *dasatinib*, *captopril*, *leflunomide*, and *dextromethorphan* targeting SRC, MMP2, PTK2B, and RAC1 hub genes, respectively. These repurposed drugs potentially target metastasis, provide pharmacodynamic insight, and offer a window of opportunity for the development of much-needed antimetastatic drugs.

## 1. Introduction

Metastasis is the lethal trait of cancer, and it is the leading cause of cancer-related mortality and morbidity [1,2]. Despite rapid advancement in our fundamental understanding of metastasis, it prevails as a major challenge in the clinical management of cancer [3]. Metastasis is a complex multistep process that begins with local tumor invasion of the surrounding stroma, followed by intravasation into the vasculature, endurance of shear stress in the vasculature, infiltration at distant sites, extravasation into the distant organ, acclimatization to the new microenvironment to form micro-metastases, and gradual proliferation into detectable macroscopic neoplasia [4]. Tumor proliferation deals with several obstacles such as low oxygen levels, immune exposure, and the pressure raised by surrounding stroma to maintain physiological integrity. During this tug of war of survival, some cancer cells gain aggressive invasive phenotypes that facilitate their detachment from the primary tumor and withstand the hostile microenvironment.

Invasive cells escape the primary tumor as a single cell or as a group, based on different stimuli, microenvironment, and external cues [5]. Notably, the invasive cells exploit a programmed process dubbed the epithelial to mesenchymal transition (EMT), a process pivotal for cell movement during embryonic development, [6,7]. In this process, epithelial cells use EMT or hybrid EMT to gain a mesenchymal phenotype by repressing E-cadherin and up-regulating N-cadherin through EMT transcription factors such as Snail, Slug, and ZEB1/2 [8,9,10,11,12]. EMT also accounts for stemness in cancer cells, enabling its transition in and out of the stem cell state [13,14]. Invasive cells also use collective migration, where cells move as a cohort, maintaining connections via cell–cell junctions and adhesion molecules [15,16]. To make a path for invasion, leader cells in the invasive front use integrins and proteases to remodel and degrade the extracellular matrix (ECM), respectively [17,18]. Cancer cells can switch between collective and single-cell dissemination during movement, based on distinct ECM density and organization [15,19].

Cancer metastasis is also considered an over-healing wound [20] as it co-opts and deregulates the cellular and molecular process of wound healing. Wound healing is crucial for repairing epithelial injuries and maintaining epithelial integrity. However, invasion in the surrounding stroma and EMT during metastasis co-opt the underlying molecular and signaling pathways of migration, proliferation, and extracellular matrix remodeling observed during the re-epithelialization process of wound repair. Cancer cells also circumvent immune surveillance by deploying wound-healing pathways [20,21,22]. Recently, a study has shown that L1CAM, which plays a key role in tissue repair, is also deployed by metastatic cells [23].

The invasive tumor cells also form an invadopodia-like protruding structure to escape primary tumor sites [24,25]. Notably, invadopodia mimic the non-invasive variant structure named podosomes, which is present in cells from monocytic myeloid lineage, endothelial cells, osteoclasts, and vascular smooth muscle cells (VSMCs). Podosomes are necessary for the normal migration of macrophages and dendritic cells via the extracellular matrix in response to interrupted tissue homeostasis [26,27]. Albeit the amount of research carried out regarding cancer metastasis, the translation of basic research into clinical practice remains a major challenge due to its complexity and prolonged time-frame to select inhibitors for preventing metastasis [28,29].

Bioinformatics can provide an opportunity to gain biological insight into metastasis by analyzing a rapidly growing wealth of information. Text-mining and citation counts of the PubMed literature database are often used to infer new information about gene-disease association, functionally related genes, and trends in medicine [30,31]. Additionally, text-mining was also applied to the healthcare domain to extract information from a vast amount of unstructured patient data [32]. Several studies have used text-mining, for instance, Jensen et al. built DISEASE, a web-based database using text-mining to correlate genes and diseases [33]. In another study, Azam et al. found global trends in prostate cancer research in genetics [34]. In the past, we have used PubMed citation counts to identify trends in hallmark-based drug discovery in breast cancer [35]. Recently, the text-mining approach was also used to identify Xerostomia-associated genes and candidate drug targets to improve clinical outcomes [36]. Currently, cancer remains the major health problem accounting for approximately 19.3 million new cases and approximately 10 million deaths worldwide, and more than 2,569,626 PubMed citations related to cancer can be used to gain biological insight into processes related to the metastasis of cancer [37].

Targeting metastasis is challenging due to its complexity and involvement of multiple steps. Therefore, exploring key genes and signaling pathways involved in key driver traits of the metastatic cascade and targeting them can be promising to impede metastasis. Based on PubMed citation counts, Meirson et al. have shown there is a gap between the growing arsenal of papers focusing on metastasis compared to available anti-metastatic drugs and therapy. Besides developing new anti-metastasis drugs, drug repurposing can be used due to its cost-effectiveness and reduced timeline to advance treatment [38]. In this study, we utilized 19,217 human genes to perform text-mining on the PubMed database to study genes involved in cancer and metastasis-associated processes such as invadopodia, motility, movement, metastasis, invasion, wound healing, EMT, and podosome. Next, by using a normalized text-mining score, we short-listed significant drivers, tumor suppressors, or oncogenes associated with each of the processes. Finally, we utilized these genes for hub gene analysis to prioritize candidate genes involved in multiple processes. These candidate genes were further used for drug re-purposing to improve treatment for metastasis prevention.

## 2. Materials and Methods

### 2.1. Preparing a List of Human Genes

A total of 20,274 and 19,834 protein-coding human genes were retrieved from BioMart and NCBI Gene, respectively. Next, a total of 19,217 commonly unique human genes from both databases were used for the text-mining analysis.

### 2.2. Text-Mining to Determine the Association of Human Genes and Biological Processes

Text-mining on the PubMed database was performed using in-house Perl script, which calculated publication counts for each human gene and biological processes such as “invadopodia”, “motility”, “movement”, “metastasis”, “cancer”, “invasion”, “wound healing”, “EMT”, and “podosome”. Double quotes were used in all our queries to obtain accurate results with an exact match. First, we counted the number of publications for each gene alone (e.g., “PTK2”), and then each gene with the name of different processes (e.g., “PTK2” AND “Invadopodia”). Further, to normalize the publication counts, the number of publication counts for each gene with each process were divided by the number of publications for each gene alone and for each process alone (i.e., [No. of papers for “PTK2” and “Invadopodia”]/[No. of papers for “PTK2” + No. of papers “Invadopodia]”) (Table S1).

### 2.3. Filtration of Cancer-Specific Text-Mining Results

Human genes were removed if normalized publication counts were not observed in at least one of the metastasis-associated processes and “cancer”. The remaining genes were further compared with the CancerMine database [39] to classify them into a driver, tumor suppressor, or oncogene. Next, the top 50 genes with the highest normalization score in each process were subjected to a Venn diagram (http://bioinformatics.psb.ugent.be/webtools/Venn/, accessed on 3 February 2022) to generate a list of non-redundant unique genes representing all the processes, which was used for downstream analysis (Table S1).

### 2.4. Correlation Analysis of Cancer-Specific Genes

Non-redundant unique genes representing all the processes were used for correlation analysis using Clustergrammer (https://maayanlab.cloud/clustergrammer, accessed on 3 February 2022) [40]. This tool uses “correlation” as the distance type and “weighted” as the linkage-type to represent a cluster of processes as a column similarity matrix, a correlation between genes and processes as a heatmap, and the clustering of genes as a gene similarity matrix (Figure S1, Table S2).

### 2.5. PPI Network, Module, and Hub Gene Analysis

STRING v11.0 (https://string-db.org, accessed on 3 February 2022) [41] was used to construct a Protein–Protein Interaction (PPI) network using a non-redundant unique gene list. *Homo sapiens* as an organism of interest was selected, the minimum required interaction score was high confidence (0.700), and the rest of the parameters were used as default. The resultant network was visualized using Cytoscape v3.8.0, Seattle, WA, USA [42]. The molecular Complex Detection (MCODE) plug-in [43] of Cytoscape was used to identify critical gene modules from the network. Critical clusters were derived using the following parameters: Find Clusters: In the whole network, Degree Cutoff: 2, Cluster Finding: Haircut, Node Score Cutoff: 0.2, K-core: 2, Maximum Depth: 100. The PPI network was further used to classify hub genes using the cytoHubba plug-in [44] of Cytoscape. The MCC (Multiple Correlation Clustering) algorithm was implemented to find the top 10% of hub genes.

### 2.6. Gene Ontology and Pathway Analysis

The identified unique genes from MCODE and MCC clustering were subjected to enrichment analysis. We used Metascape custom analysis (Minimum Overlap: 3, *p*-value Cutoff: 0.01, Minimum enrichment: 1.5) to characterize their biological processes, molecular function, cellular components, and KEGG pathway [45] (Table S3).

### 2.7. Drug-Gene Interaction

The Therapeutic Target Database (http://db.idrblab.net/ttd/, accessed on 3 February 2022) [46], DrugBank database (https://go.drugbank.com/, accessed on 3 February 2022), PubChem (https://pubchem.ncbi.nlm.nih.gov/, accessed on 3 February 2022), ClinicalTrials.gov (https://clinicaltrials.gov/, accessed on 3 February 2022), and Drugs@FDA: FDA-Approved Drugs (https://www.accessdata.fda.gov/scripts/cder/daf/index.cfm, accessed on 3 February 2022) provide existing drugs for potential therapeutic target genes. Unique genes from MCODE and MCC clustering were checked manually in the Therapeutic Target Database, DrugBank, ClinicalTrials.gov, PubChem, and FDA-approved drugs to obtain existing drugs and drugs under clinical trials for the target genes (Table S4).

### 2.8. Drug Repurposing and Connectivity Map (CMap) Analysis

The drug repurposing database CLUE (https://clue.io/, accessed on 3 February 2022) was used to advance disease treatments by listing launched drugs for potential repurposing against the unique genes from MCODE and MCC clustering. After that, drug efficacy against targets was checked based on the connectivity score (−100 to 100) present in the CLUE CMap touchstone database (Table S5).

## 3. Results

### 3.1. Association of Human Genes and Biological Processes

**Figure 1:**
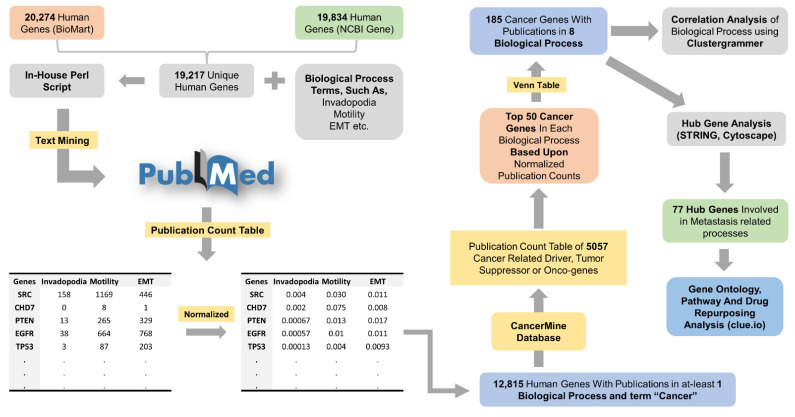
The framework of text-mining and bioinformatics analysis to study cancer metastasis-related genes.

We retrieved PubMed publication counts of 19,217 genes and each gene with cancer, and metastasis-related processes such as invadopodia, motility, movement, metastasis, invasion, wound healing, EMT, and podosome. The publication counts were then normalized, and 12,815 human genes were selected with publications in “cancer” and at least one process. A recent study has shown that almost every human gene has a connection to cancer based on present publication counts, which might create bias while interpreting results in the context of cancer-related genes [47]. To reduce the abovementioned biases, we compared these 12,815 genes with the CancerMine database and classified them into 5057 drivers, tumor suppressors, or oncogenes, depicting themselves as promising targets for therapeutic intervention. After that, the top 50 genes with the highest normalization scores in each process were subjected to a Venn diagram, and 185 non-redundant unique genes representing all the processes were selected (Figure 1).

First, we text-mined PubMed citations for each human gene and biological process related to cancer metastasis (invadopodia, motility, movement, metastasis, invasion, wound healing, EMT, and podosome). Then, PubMed citations were normalized and a total of 12,815 human genes present in at least one of the biological processes were screened for further annotation as a driver, tumor suppressor, or oncogene using the CancerMine database. Then, a total of 185 unique genes with publication in eight biological processes were selected, which was used for correlation analysis and downstream bioinformatics analysis.

### 3.2. Correlation Analysis of Cancer-Specific Genes and Process

**Figure 2:**
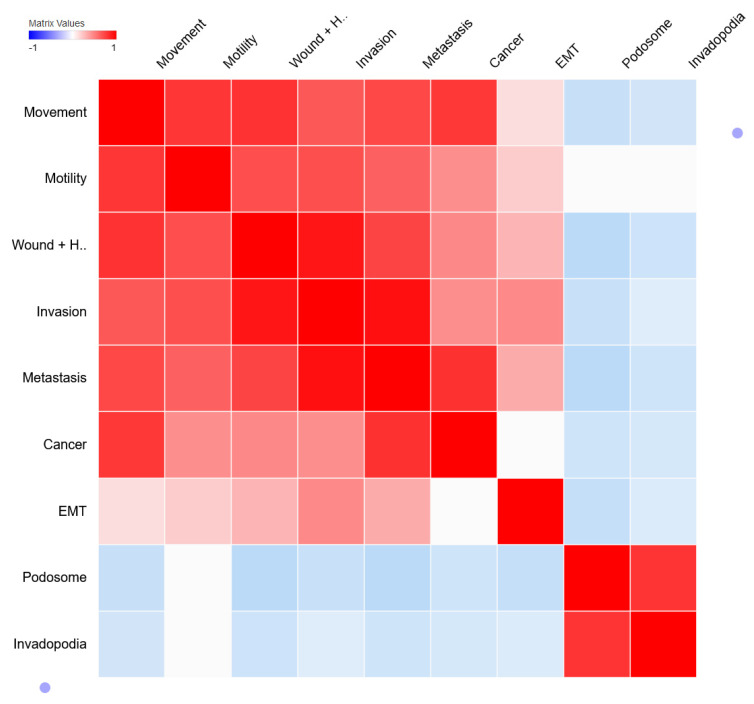
Correlation between metastasis-related processes. The column similarity matrix represents the similarity between metastasis-related processes whereas red (1), white (0), and blue (−1) represent positive, neutral, and negative correlations (measured as 1—cosine-distance).

To determine the correlation between different processes, genes with processes, and genes with genes, 185 genes with publication scores were mapped using Clustergrammer [40]. In the column similarity matrix (Figure 2), we found processes such as movement, motility, wound-healing, invasion, metastasis, and EMT (epithelial to mesenchymal transition) are associated with cancer except for invadopodia and podosome. However, we noticed an association between invadopodia and podosome. Perhaps, this could be explained by the fact that invadopodia and podosome are similar actin-rich subcellular structures, despite the former being found in cancer cells with hours of turnover time and the latter in non-cancerous cells with a few minutes of turnover time. Secondly, in scientific literature, they are frequently mentioned together or as “invadosome” [48,49,50,51]. Additionally, the gene/process association heatmap (Supplementary Figure S1) portrayed a set of genes, clustered with invadopodia and podosome but not with other processes, suggesting their role specific to invadopodia and podosome. For instance, genes such as CTTN, NCK1, SH3PXD2A, CDC42, ADAM12, SRC, RHO, PTK2, PTK2B, PXN, ADAM12, etc., are known regulators of invadopodia and podosome formation [48,49,52]. Even though genes such as SRC, RHO, PTK2, and CDC42 are also involved in other processes [53,54,55], genes specific to invadopodia and podosome formation still possess importance to be targeted as a promising therapeutic target due to their relevance to be involved in metastasis-related processes. Hence, we looked for potential genes involved in these processes as a therapeutic target.

Furthermore, understanding gene–gene associations can reveal commonly associated genes in metastasis-related biological processes. The row similarity matrix (Figure S2) portrays PubMed citations-based association between genes. We found both positive and negative associations between genes, where genes with positive associations may have some interactions (e.g., physical interactions, genetic interactions, co-expression, and co-localization) in the pathways related to these biological processes. Oppositely, genes with negative correlations should not work together to progress the metastasis. For instance, in the gene similarity matrix, Rac1 has a positive correlation with Akt1 and PTK2. Rac1 interacts with Akt1 to increase cancer cell stemness downstream of PODXL2 [56] and also enhance cell migration through the Axl/Rac1/Akt1-mediated axis [57]. PTK2 activates Rac1 to promote invasive properties in carcinoma cells [58]. As our study is based on literature citations and not on expression data, we compare our gene–gene association data with the GeneMania [59] network of 185 non-redundant unique genes to obtain the biological perspective of our text-mined data. Indeed, the resultant network (Figures S3 and S4) concurs with our gene–gene association results.

### 3.3. PPI Network, Module, and Hub Gene Analysis

**Figure 3:**
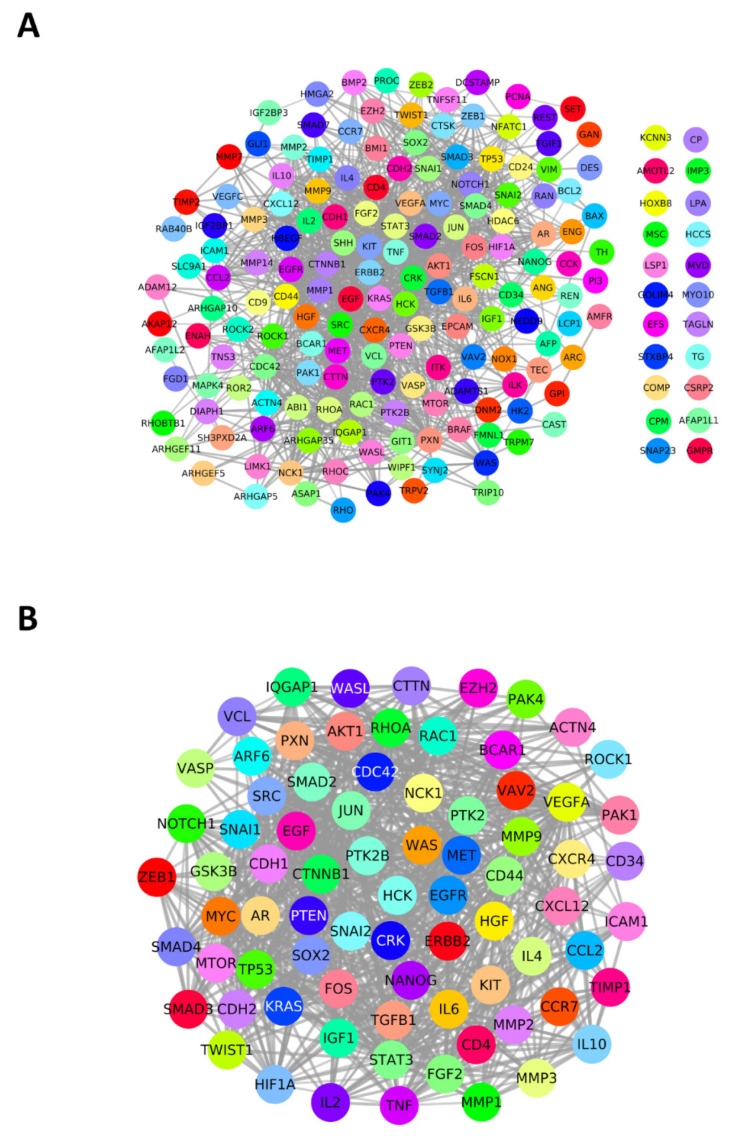
Protein–protein interaction (PPI) networks of genes and significant gene modules. (**A**) High-confidence (0.7) PPI network (STRING) of 185 unique genes involved in metastasis-related processes. (**B**) PPI network of the candidate hub genes (MCC and MCODE).

We first listed the top 50 potential genes from each process based on normalized publication scores and generated a non-redundant unique gene list of 185 genes via a Venn diagram. These 185 genes were used as seed genes to construct a PPI network with high confidence of 0.7 on the STRING database (Figure 3A). The resultant network had 185 nodes and 1326 edges. To identify potential candidate genes, the network was analyzed in two ways. First, the network was subjected to an MCODE plug-in, resulting in five clusters of connected nodes. Second, we obtained the top 10% genes, i.e., 19 hub genes from the network of 185 genes using cytoHubba MCC clustering. Altogether, 77 unique genes comprised the final list of candidate genes and were selected in the PPI network (77 nodes, 912 edges) (Figure 3B), which was used for downstream analysis such as gene enrichment and drug discovery.

### 3.4. Functional and Signaling Pathway Enrichment Analysis

**Figure 4:**
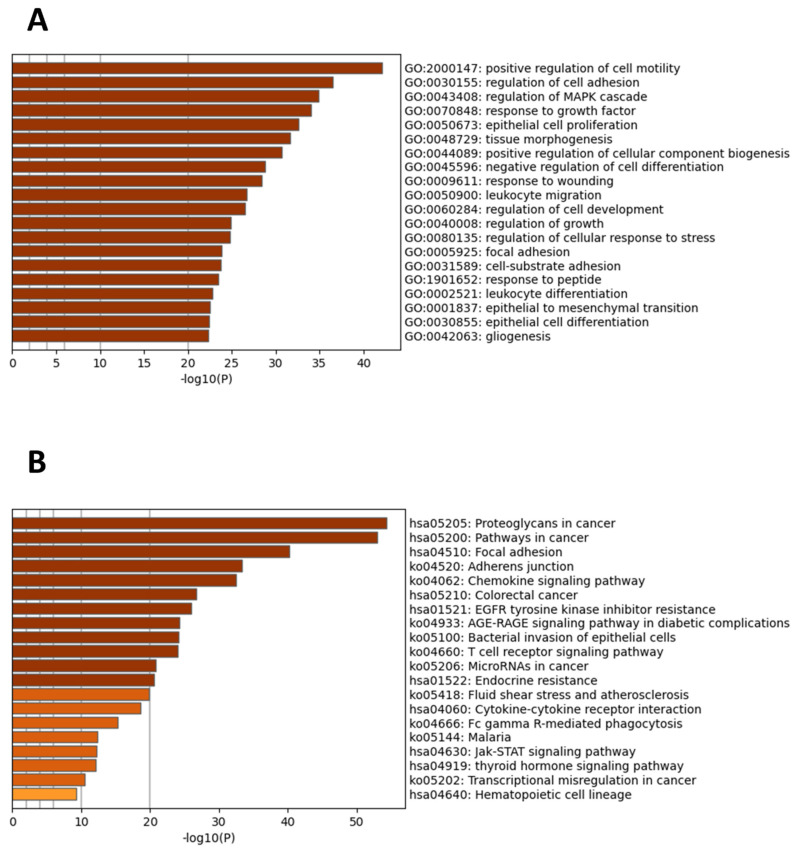
Significant gene ontology enrichment terms and enriched pathways of the candidate hub genes. (**A**) Top 20 GO terms. (**B**) Top 20 enriched KEGG pathways. GO and pathway analysis was acquired from the Metascape tool. *p* < 0.01. Dark colors represent a significant enrichment of more genes compared to lighter ones.

GO (gene ontology) term analysis of 77 candidate genes was carried out in Metascape. Significant enrichment was seen in GO terms such as the positive regulation of cell migration, regulation of cell adhesion, regulation of MAPK cascade, response to growth factor, and epithelial cell proliferation (Figure 4A). Additionally, KEGG pathway analysis revealed significantly enriched pathways. The top 7 most enriched pathways were proteoglycans in cancer, pathways in cancer, focal adhesion, the Adherens junction, the chemokine signaling pathway, colorectal cancer, and EGFR tyrosine kinase inhibitor resistance (Figure 4B).

### 3.5. Drug Target Identification and Drug Repurposing of Potential Genes

We found 50 druggable target genes of which 28 genes have drugs (approved, investigational, pre-clinical, and clinical trial) for treating cancer. Ten out of these twenty-eight genes have eleven approved drugs (Table 1), while the rest of the genes have drugs under preclinical or clinical trials (Table S4). We then used the CLUE drug repurposing database to find drugs that can be repurposed to the target genes. We found a total of 449 drugs that have the potential to be repurposed against 44 hub genes. Notably, we found a total of 50 approved drugs with connectivity scores ranging from 99.96 to 100, which can be repurposed against 19 hub genes involved in metastasis-related processes (Table S5).

## 4. Discussion

While primary tumors can often be surgically removed and account for only a small percentage of cancer-related mortality, complications associated with local invasion and distant metastasis are responsible for an estimated 90% of cancer deaths and therefore serve as a critical target for potential therapeutic intervention. Despite remarkable advances in targeted therapeutics and drugs to attenuate metastatic tumor growth, metastasis remains beyond a cure [3,60,61]. Therefore, an advanced integrative understanding of the underlying biological processes and their regulation leading to invasion and metastatic cascade initiation have paramount potential to develop targeted therapeutics for metastatic inhibition.

In this regard, we screened the candidate hub genes involved in the biological processes related to invasion and metastasis using bioinformatics methods. In total, 5057 genes were selected based on text-mining and the CancerMine database. Then, the top 50 genes according to normalized publication scores were selected from each process resulting in a unique gene list of 185 genes. These genes were subjected to a PPI network followed by MCC and MCODE clustering deriving 77 candidate hub genes. We then check for GO, KEGG pathway, and drugs available for these 77 hub genes.

GO analysis of 77 candidate hub genes shows enrichment in GO terms of the positive regulation of cell migration, regulation of cell adhesion, regulation of the MAPK cascade, response to the growth factor, epithelial cell proliferation, and response to wounding, which validate our text-mining results for each biological process. Further, pathway analysis revealed highly enriched pathways of which proteoglycans in cancer, pathways in cancer, and focal adhesion were at the top. Proteoglycans are important components of the extracellular matrix, which play a major role in ECM modulation and signal transduction by communicating with growth factors, acting as receptors, and regulating ligand–receptor interactions. Proteoglycans are well known for their involvement in multiple steps of metastasis resulting in the poor prognosis of cancer. The proteoglycans in the cancer pathway demonstrate the interaction of candidate hub genes with proteoglycans that, in turn, activate different downstream signaling pathways (such as MAPK, Wnt, PI3K-Akt, and VEGF signaling pathways) enabling tumor cell proliferation, migration, invasion, angiogenesis, growth, and survival [62,63,64], which also corroborate our screened hub gene list from text-mining data of metastasis-related biological process. Moreover, the genes are also involved in cell motility, proliferation, tissue invasion, and metastasis, evading apoptosis, sustained angiogenesis, insensitivity to antigrowth signals pathways presented in pathways in cancer, and the focal adhesion pathway in KEGG. These results also correlate with the involvement of our text-mined candidate genes in invasion and metastasis. Being involved in the multistep metastasis process, these candidate hub genes have the potential to serve as drug targets in targeted or combination therapy to achieve metastatic inhibition. We screened 10 genes with FDA-approved drugs. Src kinase is involved in metastasis by regulating invadopodia formation, cytoskeletal reorganization, and ECM adhesion contacts that influence cell motility. It is also induced by TF Snail to promote the collective migration of cancer cells [15,25]. Moreover, Lilly et al. have shown that the proteoglycans hyaluronan-CD44/c-Src/Twist axis takes part in breast tumor cell invasion. Thus Src and CD44 can serve as a good therapeutic target for metastatic inhibition [65]. We found *bosutinib* and *dasatinib* as approved drugs for Src. However, the drug (A-6) for CD44 to treat macular degeneration disease is presently under phase II clinical trial, which, after approval, might be repurposed for cancer in the future. Presently, the Clue repurposing database has shown that hyaluronic acid used for osteoarthritis and interstitial cystitis can be used to target CD44. FGF2 and CCND1 have been reported to play role in migration and invasion in papillary thyroid carcinoma [66]. FGF2 and EGF also promote metastasis by activating the ERK/MAPK signaling pathway. FGF2 does not yet have any approved drugs used in cancer treatment. However, Acitretin used in psoriasis, Niclosamide used for treating tapeworm infection, and Sirolimus used in organ rejection or lymphangioleiomyomatosis treatment can be repurposed against FGF2. Moreover, TP53, MMP9, and CTNNB1 are involved in promoting metastasis through migration, invasion, EMT, and wound healing [67,68,69,70]. TP53 has the drug *Advexin* under clinical trial for treating different types of cancer. *Mepacrine* is used to treat giardiasis. *GS-5745* and anti-metastatic agent *Marimastat*, which target MMP9, are currently under clinical investigation. *Captopril* used to treat hypertension, congestive heart failure, myocardial infarction, diabetes mellitus, and diabetic nephropathy can be used to target MMP9. Further, to our surprise, we found that *Aspirin* (analgesic), *curcumin*, and *urea* can be repurposed against TP53, MMP9, and CTNNB1, respectively. We also noted *Eugenol* (Clove oil), a toothache medication, and the contraceptive *Norgestrel* can be repurposed against the gene AR (Androgen Receptor).

Recently, metastasis-free survival has been approved by the Food and Drug Administration (FDA) as a new primary endpoint in clinical trials [71]. This will direct the focus of research and development towards the discovery of antimetastatic treatment first, through the discovery of a new drug that can be used alone or in combination with standard cytotoxic therapy, and second, by repurposing approved drugs. In this study, we have screened genes from different biological processes involved in the initiation of metastasis, looked for druggable genes, and repurposed drugs that might bring value to advance treatment for metastatic inhibition. We also used the GeneMania web interface to gain insight into the functional interaction between candidate hub genes (Figure S5). Physical interaction (44.04%) followed by co-expression (27.34%) scored high, suggesting the potential of this PPI network to be explored as a target for drug discovery in the future. Further, critical candidate hub genes can be used as a target to develop migrastatics drugs [72] in the future to impede metastasis, the deadliest trait of all malignant cancer.

## 5. Conclusions

The text-mining approach has been used to sift through a vast amount of PubMed literature to discover the association between biological processes involved in metastasis and further gene prioritization. Here, we found that genes and biological processes involved in metastasis were significantly correlated. We also looked for hub genes involved in these processes to find a druggable target. Further, we looked for drugs that have the potential to be repurposed against hub genes involved in metastasis to improve treatment outcomes. The genes and drugs we have shown were only preliminary studied and hence require validation based on experiments and computer simulation. Recent studies have used Bidirectional Encoder Representations from Transformers (BERT), a Natural Language Processing (NLP) approach, to identify genes from clinical databases and for disease prediction from clinical databases [73,74]. Hence, future work implementing BERT to obtain information from big clinical and omics data could be useful to predict candidate genes for drug repurposing.

In terms of caveats, our study is based on literature citations, and potential pitfalls include over-citations and under-citations. Furthermore, we do not differentiate between genetics, epigenetics, and post-translational modifications.

## Figures and Tables

**Table 1:** List of approved drugs for candidate hub genes based on TTD database, DrugBank database, PubChem, ClinicalTrials.gov, and Drugs@FDA: FDA-Approved Drugs.

No.	Gene	Drug	Drug Type	Mechanism of Action	Disease
1	EGFR	Afatinib	Small molecule	Inhibitor	HER2/NEU overexpressing breast cancer
		Osimertinib	Small molecule	Inhibitor	metastatic non-small cell lung cancer
2	MET	Capmatinib	Small molecule	Inhibitor	non-small cell lung cancer
3	MTOR	Temsirolimus	Small molecule	Inhibitor	renal cell carcinoma
4	EZH2	Tazemetostat	Small molecule	Inhibitor	Follicular lymphoma
5	KIT	Ripretinib	Small molecule	Inhibitor	gastrointestinal stromal tumor
6	CXCR4	Plerixafor	Small molecule	Antagonist	non-Hodgkin’s lymphoma, multiple myeloma
7	IL2	Aldesleukin	Protein Based Therapies	Agonist, modulator	renal cell carcinoma
8	SRC	Bosutinib	Small molecule	Inhibitor	hematologic malignancy
		Dasatinib	Small molecule	Inhibitor	hematologic malignancy
9	HCK	Bosutinib	Small molecule	inhibitor	hematologic malignancy
10	ERBB2	Trastuzumab	monoclonal antibody	Antagonist/Inhibitor	HER2-positive breast, gastroesophageal, and gastric cancers

## Supplementary Materials

The following supporting information can be downloaded at: https://www.mdpi.com/article/10.3390/jcm11082130/s1. Figure S1: The heatmap represents the association between genes and metastasis-related processes. Figure S2: Gene-gene association. The row similarity matrix represents a correlation between genes. Figure S3. GeneMania network 185 non-redundant unique genes, presenting interactions between genes. Figure S4. Example of GeneMania network 185 non-redundant unique genes, highlighting interactions between Rac1 with other genes. Figure S5. GeneMania network 77 candidate hub genes, presenting interactions between genes. Table S1: Text-mining results for association between human genes and metastasis related biological processes, and, cancer-specific filtered text-mining results. Table S2: Correlation analysis of cancer metastasis related genes and biological processes using Clustergrammer. Table S3: Gene ontology and pathway analysis results of cancer-specific unique genes. Table S4: List of manually validated pre-clinical drugs to target hub genes. Table S5: List of FDA approved/pre-clinical drugs, also validated on cancer-cell lines (CMap Score) for drug repurposing against hub genes.
