# Supplementary material for: Text-Mining Approach to Identify Hub Genes of Cancer Metastasis and Potential Drug Repurposing to Target Them
Source: J Clin Med. 2022 Apr 11;11(8):2130. doi: 10.3390/jcm11082130 (PMC9029557; doi:10.3390/jcm11082130)
Supplement: Supplementary file 1 [file jcm-11-02130-s001.zip › Supporting_Information_Legends.pdf]

Supplementary Information:

Figure S1. The heatmap represents the association between genes and metastasis-related processes. Red (1) and white (0) cells in the matrix represent positive and neutral correlation values in the matrix.

Figure S2. Gene-gene association. The row similarity matrix represents a correlation between genes. Red (1), white (0), and blue (-1) represents positive, neutral, and negative correlation (measured as 1 – cosine-distance of association).

Figure S3. GeneMania network 185 non-redundant unique genes, presenting interactions between genes. Different network colors represent different associations, such as physical interaction, co-expression, genetic interaction, predicted association, co-localization, shared pathway, and shared protein domain homology.

Figure S4. Example of GeneMania network 185 non-redundant unique genes, highlighting interactions between Rac1 with other genes.

Figure S5. GeneMania network 77 candidate hub genes, presenting interactions between genes. Different network colors represent different associations, such as physical interaction, co-expression, genetic interaction, predicted association, co-localization, shared pathway, and shared protein domain homology.

Table S1: Text-mining results for association between human genes and metastasis related biological processes, and, cancer-specific filtered text-mining results.

Table S2: Correlation analysis of cancer metastasis related genes and biological processes using Clustergrammer.

Table S3: Gene ontology and pathway analysis results of cancer-specific unique genes.

Table S4: List of manually validated pre-clinical drugs to target hub genes.

Table S5: List of FDA approved/pre-clinical drugs, also validated on cancer-cell lines (CMap Score) for drug repurposing against hub genes.
