# Supplementary figures and images for: Text-Mining Approach to Identify Hub Genes of Cancer Metastasis and Potential Drug Repurposing to Target Them
Source: J Clin Med. 2022 Apr 11;11(8):2130. doi: 10.3390/jcm11082130 (PMC9029557; doi:10.3390/jcm11082130)

Matrix Values

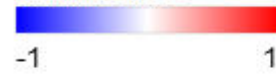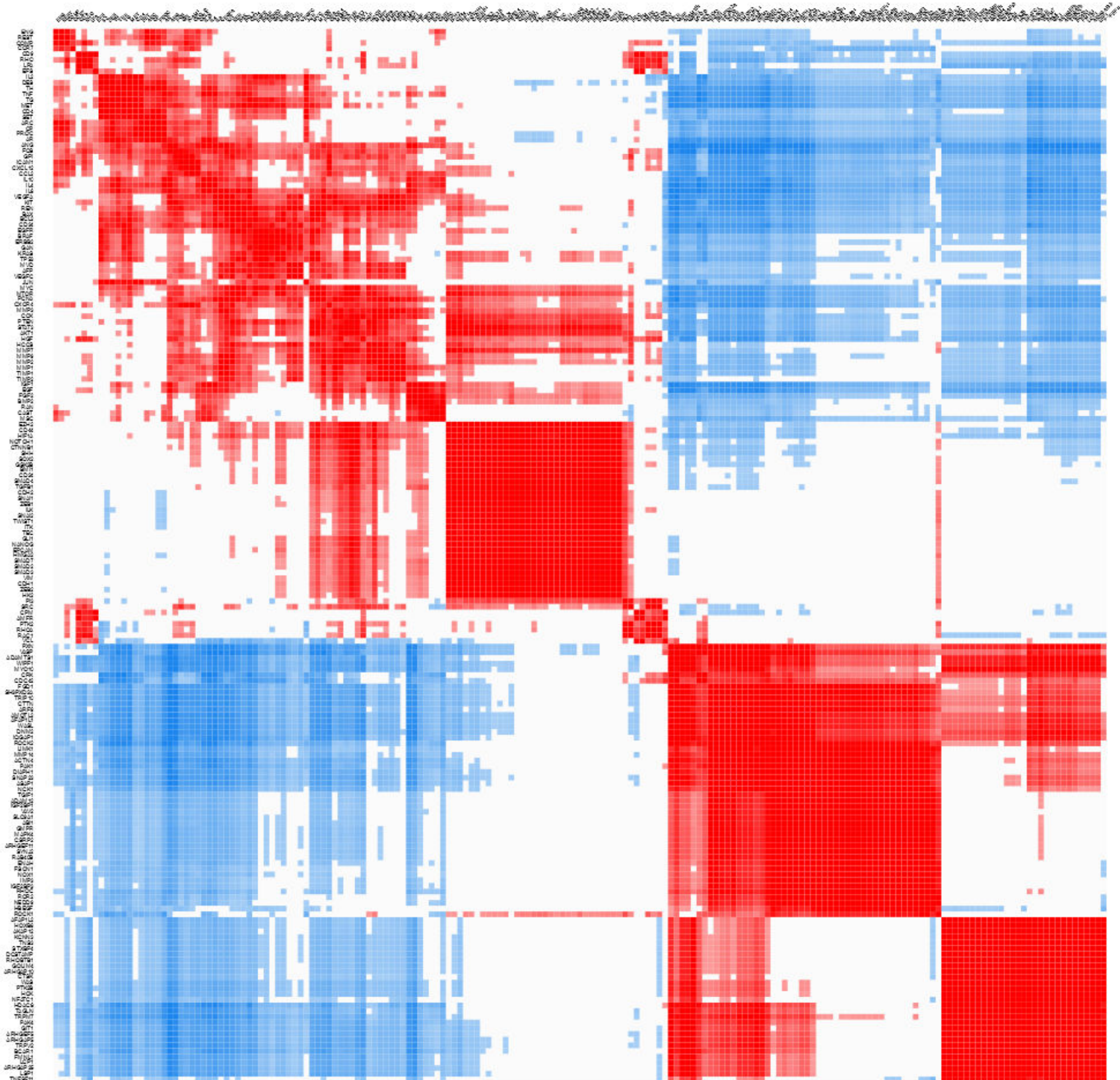

Supplement: Supplementary file 1 [file jcm-11-02130-s001.zip › Figure_S2.pdf]

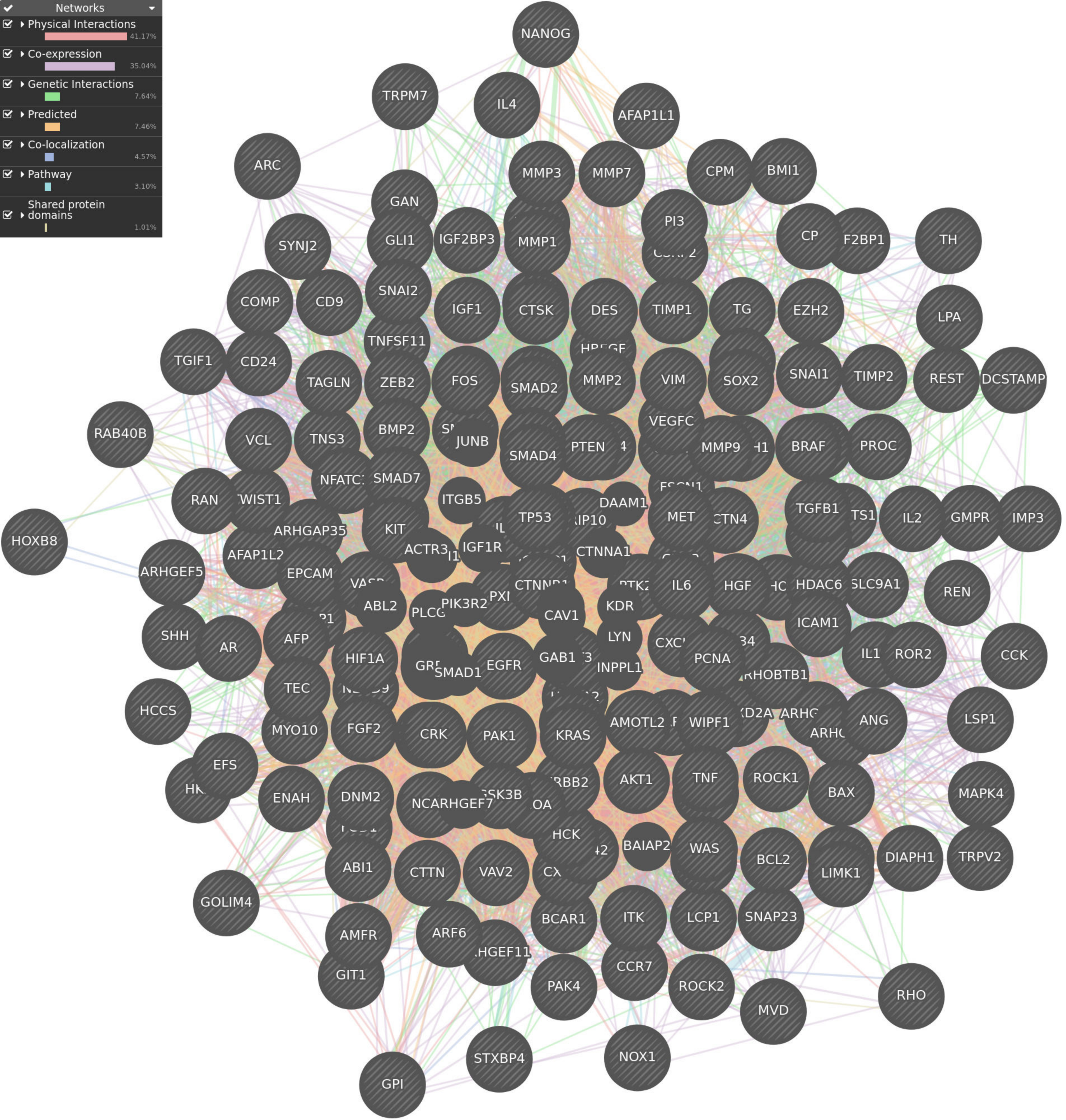

Supplement: Supplementary file 1 [file jcm-11-02130-s001.zip › Figure_S3.pdf]

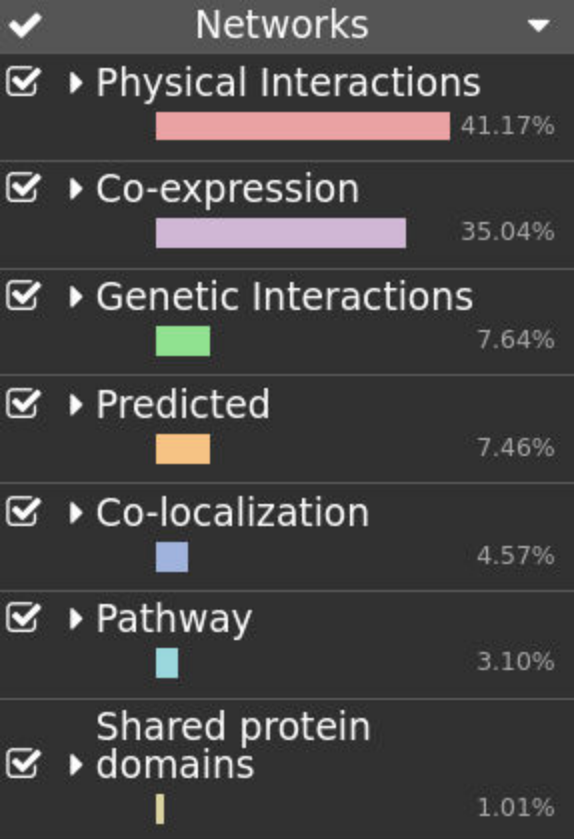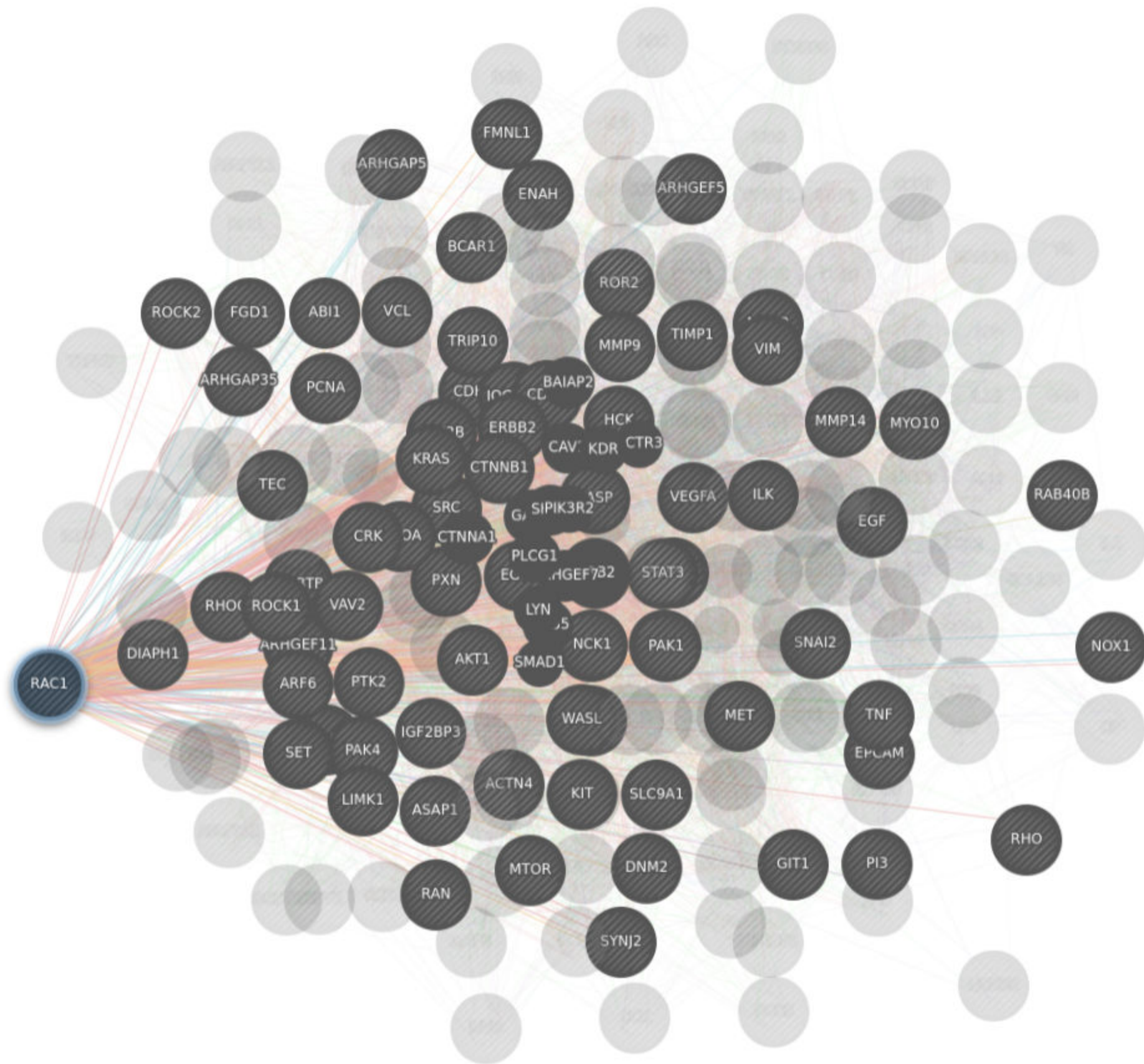

Supplement: Supplementary file 1 [file jcm-11-02130-s001.zip › Figure_S4.pdf]

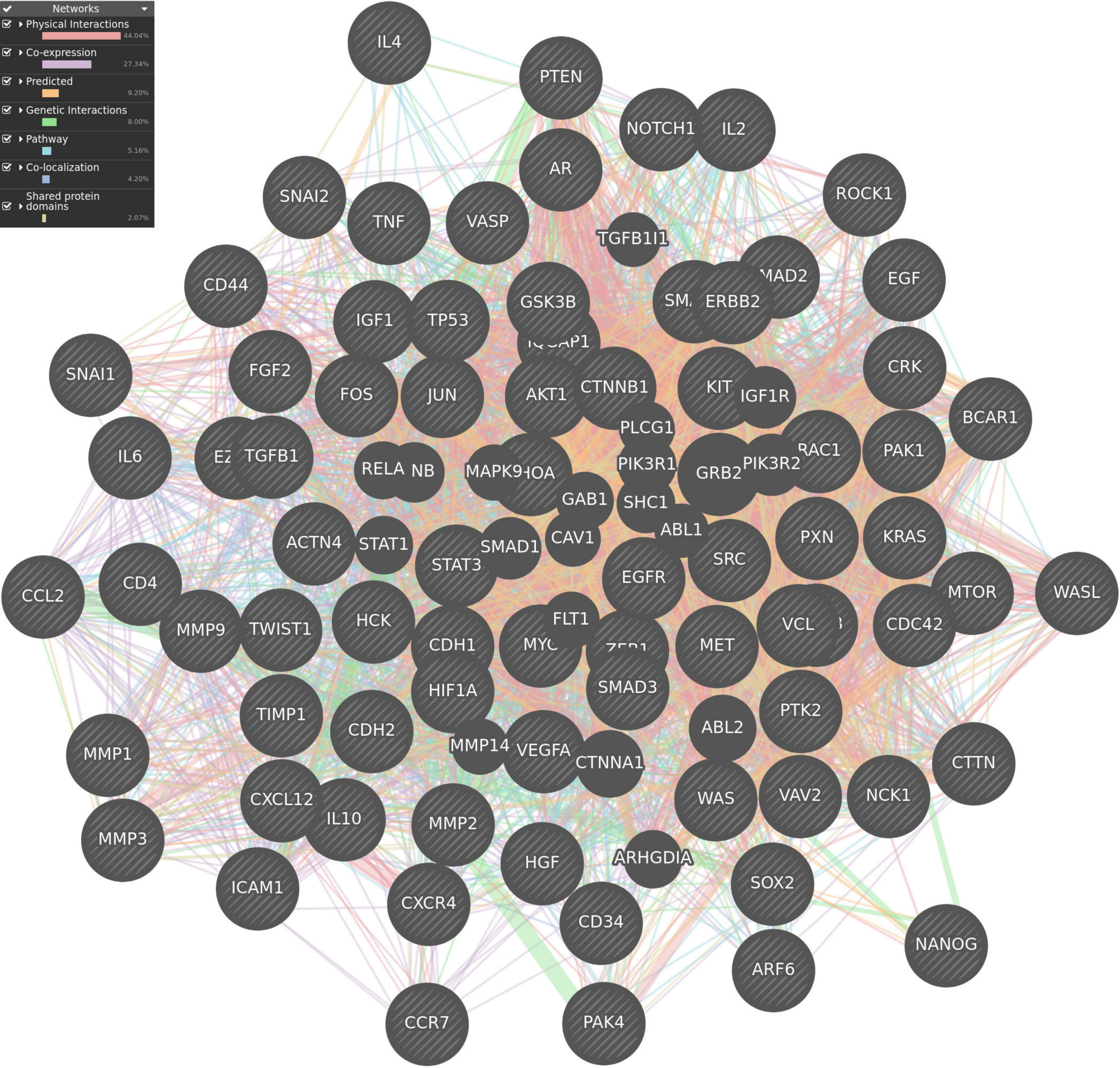

Supplement: Supplementary file 1 [file jcm-11-02130-s001.zip › Figure_S5.pdf]
